# Cellular and humoral immune responses and breakthrough infections after three SARS-CoV-2 mRNA vaccine doses

**DOI:** 10.3389/fimmu.2022.981350

**Published:** 2022-08-17

**Authors:** Patricia Almendro-Vázquez, Marta Chivite-Lacaba, Alberto Utrero-Rico, Cecilia González-Cuadrado, Rocio Laguna-Goya, Miguel Moreno-Batanero, Laura Sánchez-Paz, Joanna Luczkowiak, Nuria Labiod, María Dolores Folgueira, Rafael Delgado, Estela Paz-Artal

**Affiliations:** ^1^ Instituto de Investigación Sanitaria Hospital 12 de Octubre (imas12), Madrid, Spain; ^2^ Department of Immunology, Hospital Universitario 12 de Octubre, Madrid, Spain; ^3^ Centro de Investigación Biomédica en Red (CIBER) de Enfermedades Infecciosas (CIBERINFEC – Instituto de Salud Carlos III), Madrid, Spain; ^4^ Department of Microbiology, Hospital Universitario 12 de Octubre, Madrid, Spain; ^5^ Department of Medicine, Medical School, Universidad Complutense de Madrid, Madrid, Spain; ^6^ Department of Immunology, Ophthalmology and ENT, Medical School, Universidad Complutense de Madrid, Madrid, Spain

**Keywords:** SARS-CoV-2, COVID-19, vaccination, third dose, immune response, breakthrough infection, hybrid immunity

## Abstract

**Background:**

SARS-CoV-2 vaccination has proven the most effective measure to control the COVID-19 pandemic. Booster doses are being administered with limited knowledge on their need and effect on immunity.

**Objective:**

To determine the duration of specific T cells, antibodies and neutralization after 2-dose vaccination, to assess the effect of a third dose on adaptive immunity and to explore correlates of protection against breakthrough infection.

**Methods:**

12-month longitudinal assessment of SARS-CoV-2-specific T cells, IgG and neutralizing antibodies triggered by 2 BNT162b2 doses followed by a third mRNA-1273 dose in a cohort of 77 healthcare workers: 17 with SARS-CoV-2 infection prior to vaccination (recovered) and 60 naïve.

**Results:**

Peak levels of cellular and humoral response were achieved 2 weeks after the second dose. Antibodies declined thereafter while T cells reached a plateau 3 months after vaccination. The decline in neutralization was specially marked in naïve individuals and it was this group who benefited most from the third dose, which resulted in a 20.9-fold increase in neutralization. Overall, recovered individuals maintained higher levels of T cells, antibodies and neutralization 1 to 6 months post-vaccination than naïve. Seventeen asymptomatic or mild SARS-CoV-2 breakthrough infections were reported during follow-up, only in naïve individuals. This viral exposure boosted adaptive immunity. High peak levels of T cells and neutralizing antibodies 15 days post-vaccination associated with protection from breakthrough infections.

**Conclusion:**

Booster vaccination in naïve individuals and the inclusion of viral antigens other than spike in future vaccine formulations could be useful strategies to prevent SARS-CoV-2 breakthrough infections.

## Introduction

Vaccination against SARS-CoV-2 has been revealed as the most effective measure to control the COVID-19 pandemic preventing infection and especially severe disease (1). The 2-dose mRNA vaccines BNT162b2 (Pfizer-BioNTech) and mRNA-1273 (Moderna) encoding a stabilized version of the full-length spike protein have been broadly administered after showing safety and a 95% efficacy in preventing symptomatic disease in clinical trials (2, 3). These mRNA vaccines elicit an early and potent humoral immune response (4–6), that begins to decline after 1 month post-vaccination (7, 8). However, circulating antibodies persist for at least 6 months (8–10) and their neutralizing activity has been detected up to 8 months after vaccination (11). Moreover, the efficacy of the vaccine in preventing hospital admissions and deaths is maintained beyond six months post-vaccination (12, 13). Yet, some studies report that decreased antibody levels may be associated with an increased rate of infections (14, 15).

Immunological studies of SARS-CoV-2 infection highlight the role of specific T cells in disease control (16–18) and support that only measuring specific IgG may underestimate protection against COVID-19 (19, 20). mRNA vaccination has also been shown to develop spike-specific T cells (5, 19, 21, 22) that can contribute to protection upon exposure to the virus. Nevertheless, the durability and magnitude of memory T cells after vaccination remains poorly understood since only a handful of studies have studied the maintenance of T cell response up to six months post-vaccination (4, 23–25).

The emergence of several SARS-CoV-2 variants and the increased transmission have promoted the application of third vaccine booster dose strategies in different countries. However, information on how preexisting SARS-CoV-2 humoral and cellular immunity are boosted by a third mRNA vaccination remains limited. Recent studies have shown improved effectiveness of a third dose in preventing SARS-CoV-2 infection and severe illness (26–28) and an increase in antibodies levels (29, 30) and neutralization efficiency (31). Yet, data on the real-world T cell immunity dynamic after third doses of COVID-19 mRNA vaccine are still very scarce (32–34).

Here we show the first longitudinal analysis including the cellular, IgG and neutralizing antibody responses elicited by the 2-dose BNT162b2 vaccination plus a third mRNA-1273 boost dose in 77 healthcare workers, 17 of whom had suffered SARS-CoV-2 infection prior to the first vaccine dose. SARS-CoV-2-specific T cells remained stable 3 to 6 months after 2-dose mRNA vaccination, whereas antibodies and their neutralizing activity gradually declined. We also describe that SARS-CoV-2 recovered individuals maintained higher levels of specific T cells and total and neutralizing antibodies than naïve individuals. In addition, administration of the third vaccine dose boosted cell- and antibody-mediated immunity, especially T cells in recovered subjects and neutralizing antibodies in naïve subjects. Finally, we describe the association between high peak levels of T cells and neutralizing antibodies post-vaccination and protection from breakthrough infections. These analyses provide insights into mRNA vaccine-induced immunogenicity and may be relevant for future vaccine strategies, including recommendations and target populations for booster doses.

## Materials and methods

### Study design and sample collection

**Figure 1:**
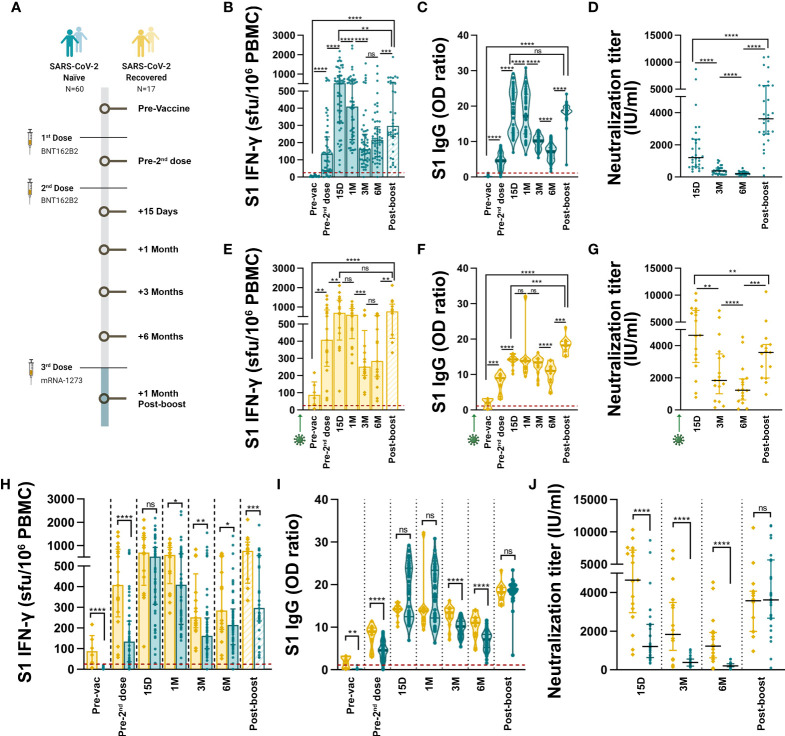
Development and maintenance of SARS-CoV-2-specific cellular and humoral immunity after 3 mRNA vaccine doses. **(A)** Study design and cohorts, samples were collected pre-vaccine, 21 days after the first dose, 15, 30, 90 and 180 days after the second dose and 30 days after third boost dose. **(B)** Dynamics of vaccine-triggered S1-IFN-γ-producing T cell response in SARS-CoV-2 naïve individuals (in blue). Data is represented as spot forming unit (sfu) per million PBMC. Dashed lines represent the positivity cut-off established by using a non-infected control group: >25 sfu/10^6^ PBMC. **(C)** anti-S1 IgG levels elicited by mRNA vaccination in SARS-CoV-2 naïve individuals. Dashed line represents the established cut-off of positivity: OD ratio ≥1.1. **(D)** Vaccine-triggered neutralizing activity in SARS-CoV-2-naïve serum samples, represented as International Units per ml (IU/ml). **(E–G)** Dynamics of S1-IFN-γ-producing T cell **(E)**, anti-S1 IgG **(F)** and neutralizing antibody **(G)** responses elicited by mRNA vaccination in SARS-CoV-2 recovered individuals (in yellow). **(H–J)** Comparison of S1 T cell **(H)**, anti-S1 IgG **(I)** and neutralizing **(J)** responses between SARS-CoV-2 naïve and recovered individuals. Green arrows represent the time of SARS-CoV-2 infection. Horizontal bars and whiskers represent median values and interquartile ranges, respectively. The significance between groups was determined using Mann Whitney, Wilcoxon signed rank or Kruskal-Wallis tests, ns: not statistically significant, *p<0.05, **p<0.01, ***p<0.001, ****p<0.0001.

Seventy-seven healthcare workers undergoing COVID-19 vaccination were recruited at Hospital Universitario 12 de Octubre (Madrid, Spain). Participants received the two 30 µg dose, 21 days apart, BNT162b2 vaccination schedule in early 2021, and after 10 months they received the 50-µg mRNA-1273 boost, in December 2021. Samples were longitudinally collected at 7 time points: pre-vaccine baseline, 21 days after the first BNT162b2-dose, 15 days, 1, 3 and 6 months after the second BNT162b2-dose and 30 days after mRNA-1273 third boost dose. The subjects were further stratified on the basis of SARS-CoV-2 infection prior to vaccination confirmed by either positive RT-PCR or S1 domain of the Spike glycoprotein (S1), Nucleocapsid (N) and/or Membrane (M) SARS-CoV-2-specific T cells above the positivity threshold by fluorospot (60 SARS-CoV-2-naïve; 17 SARS-CoV-2-recovered) (**Figure 1A**). Vaccination side effects after the three vaccine doses were collected using a standardized questionnaire. The Institutional Review Board approved the study (21/039 and 21/056).

### FluoroSpot assay

Freshly isolated peripheral blood mononuclear cells (PBMCs) were seeded in duplicate at 300,000 cells/well in IFN-γ IL-2 FluoroSpot™ plates (MabTech). Cells were supplemented with 15-mer overlapping peptides covering the S1 domain of the Spike glycoprotein (SARS-CoV-2 S1 scanning pool, MabTech), the Nucleocapsid protein (Epitope Mapping Peptide Set [EMPS] SARS-CoV-2 NCAP-1, JPT), and the Membrane protein (EMPS SARS-CoV-2 VME1, JPT) at a final concentration of 1 µg/mL. Assays were incubated for 16-18 hours at 37°C. Spots were counted using automated IRIS™ FluoroSpot Reader System (MabTech). Results were expressed as IFN-γ- or IL-2-producing spot forming units (sfu) per 10^6^ PBMCs. Reponses were considered positive if the results were at least three times higher than the mean of the negative control and >25 IFN-γ sfu/10^6^ PBMCs for S1, >14 IFN-γ sfu/10^6^ PBMCs for N, and >21 IFN-γ sfu/10^6^ PBMCs for M proteins, as previously published (19, 35). Apart from the sfu, which correspond to the frequency of antigen-specific T cell clones, the relative spot volume (RSV), which represented the amount of secreted IFN-γ per spot, was also obtained.

### Anti-S1 IgG detection by ELISA

Serum SARS-CoV-2 IgG antibodies targeting the S1 protein were detected with the Euroimmun Anti-SARS-CoV-2 ELISA (Euroimmun AG, Lübeck, Germany) according to manufacturer’s instructions. Results were evaluated semi-quantitatively by calculating the ratio of the OD value of the sample over the OD value of the calibrator (relative OD), with the following cut-off values: OD ratio <1.1: negative; and OD_ratio ≥1.1: positive.

### SARS-CoV-2 neutralization assay

SARS-CoV-2-pseudotyped rVSV-luc were produced as previously published (11, 36, 37). Serum samples were tested at dilutions 1:80, 240, 720, 2160, 6480. SARS-CoV-2-pseudotyped rVSV-luc was normalized for infectivity to a MOI of 0.5 and incubated with the serum samples at 37° C for 1 h. Next, Vero E6 cells were seeded onto the virus-serum mixture and incubated at 37°C for 24h. Cells were then lysed and assayed for luciferase expression. Neutralizing titer 50 (NT50) was calculated using a nonlinear regression model fit with settings for log agonist versus normalized response curve. Neutralization potency of serum samples were calibrated using the WHO International Standard 20/136, which was assigned 813 International Units per ml [IU/ml]. Calibrated NT50 in IU/ml for each serum sample was calculated as the observed NT50 titers multiplied by the calibration factor (assigned as 0.756), which is estimated as 813 IU/ml divided by NT50 (tested as 1:1075) of the 20/136 standard (11, 36). The cut-off titer for neutralization positivity was 45 IU/ml (38).

### Statistical analysis

Quantitative data were shown as the median with IQR, and qualitative variables were expressed as absolute and relative frequencies. Non-parametric Mann-Whitney U or Wilcoxon signed-rank tests were applied for comparison within two groups, when necessary. Kruskal-Wallis test was used to compare three or more unmatched groups. Correlations between continuous variables were evaluated using Spearman’s rank test. Grouping of individuals was done according to mean SFU and neutralization titer. Differences were considered statistically significant when p<0.05. Statistical analysis was performed using GraphPad Prism version 8.0 software (GraphPad Software Inc, LaJolla, CA) and R software v4.1.1.

## Results

### Cohort design and participants

Cellular and humoral SARS-CoV-2-specific immune responses were prospectively analysed in 77 healthcare workers undergoing vaccination, comprising a total of 539 longitudinal samples studied (**Figure 1A**). The median age of the cohort was 39 years old (interquartile range [IQR] 22-64 years) and 57/77 (74%) participants were females, with no relevant comorbidities. Based on SARS-CoV-2 infection prior to vaccination, the cohort included 60 SARS-CoV-2 naïve and 17 recovered individuals. All recovered subjects had been infected by the ancestral Wuhan SARS-CoV-2 strain during the first pandemic wave in Spain. They had been either asymptomatic or suffered mild COVID-19 (WHO ordinal scale of 0-2). The median time from symptom onset to administration of the first BNT162b2-dose was 285 days ([IQR] 272-298 days). There were no differences in sex or age between naïve and recovered individuals. Vaccination side effects were more frequent among subjects with prior SARS-CoV-2 infection compared to naïve subjects (**Figure S1**), and increased their frequency with subsequent vaccine doses (median [IQR] number of side effects after the first, second and third doses were 2 [2-3], 4 [2-6] and 5 [3-7], respectively).

### Vaccine-triggered adaptive immune response dynamics

We studied the development and maintenance of SARS-CoV-2-specific T cells, IgG, and neutralizing antibodies after vaccination in SARS-CoV-2 naïve and recovered individuals. Among naïve subjects, the peak S1-specific cellular response was achieved 15 days after the second vaccine dose, then it decreased steadily and finally reached a plateau at month 3, remaining stable up to month 6 post-vaccination (**Figure 1B**). All vaccinees except one remained above the positivity threshold for cellular response during this period. The administration of the third booster dose significantly increased the number of S1-specific T cells compared to 6 months post-vaccination (**Figure 1B**), although, the magnitude of this response did not reach peak levels. Regarding the humoral response, peak levels of S1-specific IgG antibodies were also detected 15 days after the second dose, and then they gradually declined over the next 6 months although remained detectable in all subjects (**Figure 1C**). The third vaccine dose boosted anti-S1 IgG to levels comparable to the peak response (**Figure 1C**). Similarly, the maximum neutralizing response against SARS-CoV-2 elicited by 2 vaccine doses was observed 15 days after the second dose and it experienced a remarkable reduction over the course of the next 6 months (**Figure 1D**). All vaccinees except one remained positive for neutralization, although the levels of neutralization 6 months after the second dose were only slightly above the 45 IU/ml positivity threshold. Among naïve individuals the third vaccine dose elicited the highest levels of neutralizing antibodies, with a 20.9-fold increase compared to the previous measurement (**Figure 1D**). Anti-S1 IgG positively correlated with the neutralizing activity of serum samples at all time points (**Figure S2**). The development of IL-2-producing and IFN-γ+IL-2 double positive S1-specific T cells followed a dynamic similar to that of IFN-γ-producing T cells (**Figures S3A, B**), although 3 and 6 months after the second dose the number of IL-2-producing clones significantly exceeded that of IFN-γ (**Figure S3C**).

Similar to naïve individuals, in SARS-CoV-2 recovered subjects the maximum cellular response to vaccination was reached 2 weeks after the second dose, it declined up to month 3 and then remained stable up to month 6 (**Figures 1E**, **S3D, E**). During this period, there were more IFN-γ-producing T cells than IL-2 (**Figure S3F**). The third vaccine dose significantly enhanced the S1-specific T cell response which reached peak levels (**Figure 1E**). Similarly, anti-S1 IgG levels reached their peak 2 weeks after the second dose and then experienced a progressive decline up to month 6, although all subjects remained S1-IgG-positive (**Figure 1F**). After the third dose the anti-S1 IgG antibodies were the highest. The neutralizing activity followed a similar profile to that of total antibodies, and despite the decline over time, neutralizing antibodies remained well above the positivity threshold 6 months after vaccination (**Figure 1G**). The third vaccine administration boosted the neutralizing activity with a 2.3-fold increase, which nevertheless did not reach the maximal level observed 15 days post-second dose. As in naïve individuals, anti-S1 IgG positively correlated with the neutralizing activity of serum samples at all time points (**Figure S2**).

### Distinct patterns and maintenance of response in SARS-CoV-2 naïve and recovered individuals

mRNA vaccines induced robust circulating cellular and antibody responses specific to the S1 SARS-CoV-2 protein. However, these responses showed different dynamics depending on whether or not individuals had COVID-19 prior to vaccination. Although the peak T cell response elicited 2 weeks after the second dose was similar in both groups (**Figures 1H**, **S3G, H**), the maintenance of IFN-γ-producing T cell responses thereafter was significantly higher in SARS-CoV-2 recovered than in naïve individuals, both the number of specific T cells and the amount of IFN-γ released by each T cell clone (**Figures 1H**, **S3I**). After the third dose, the T cell response developed in subjects with prior COVID-19 was significantly higher both in the number of S1-specific clones (**Figure 1H**) and in the amount of IFN-γ secreted per clone (**Figure S3I**) compared to naïve individuals.

Humoral response paralleled T cell behaviour, since anti-S1 IgG peak levels were similar at 2 weeks post-second vaccine dose but the decline in antibody levels was significantly lower in SARS-CoV-2 recovered compared to naïve individuals over the course of the next 6 months (**Figure 1I**). The administration of the third vaccine dose elicited similar anti-S1 IgG levels in both groups (**Figure 1I**). Two-dose vaccination in SARS-CoV-2 recovered individuals induced a much higher level of neutralizing antibodies as compared with naïve individuals up to 6 months post-vaccination (**Figure 1J**). Three vaccine doses were required to develop a similar neutralizing activity in both cohorts (**Figure 1J**). The boost in neutralizing capacity triggered by the third dose was much higher in naïve than in recovered individuals (20.9- versus 2.3-fold increase).

We found no association between age and strength of the cellular or humoral immune response as measured at month 6 after 2-dose vaccination, either in SARS-CoV-2 naïve or recovered individuals (**Figures S4A–C**). Finally, significant positive correlations between the number of side effects and the magnitude of both T cell and antibody responses were exclusively found in SARS-CoV-2 naïve individuals after the second vaccine dose (**Figures S4D–I**).

### Breakthrough SARS-CoV-2 infections after mRNA vaccination

**Figure 2:**
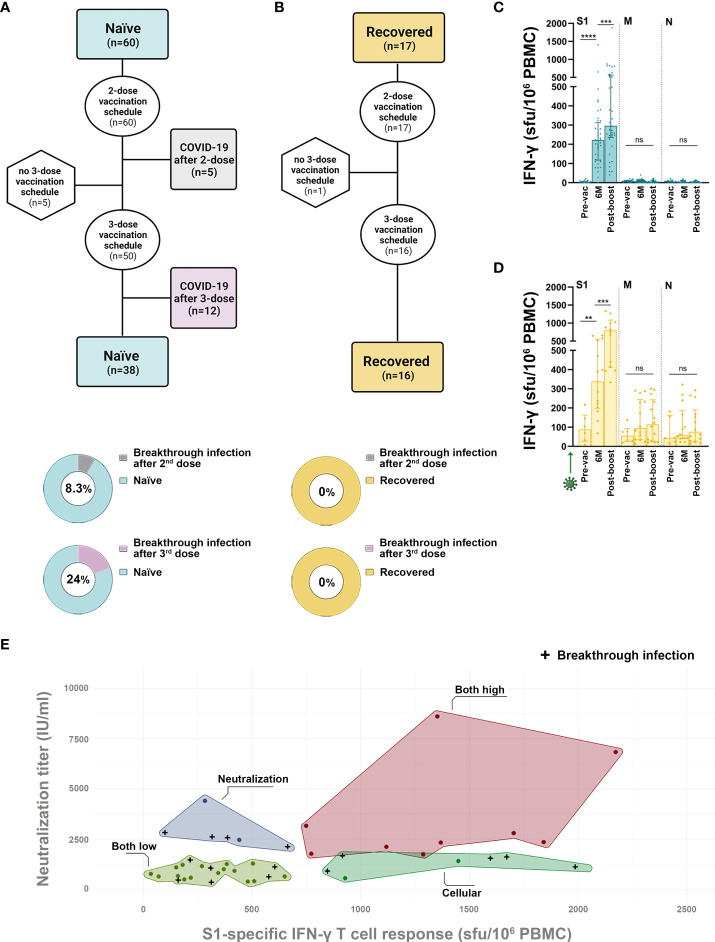
Factors associated with protection against breakthrough infection after SARS-CoV-2 vaccination. **(A, B)** Flowchart of SARS-CoV-2 naïve **(A)** and recovered **(B)** individuals included in the study and the frequency of SARS-CoV-2 infection reported after the second or third vaccine dose. **(C, D)** SARS-CoV-2-specific IFN-γ-producing T cell responses reactive to the S1, M and N proteins in subjects who remained SARS-CoV-2 naïve during follow-up **(C)** and in recovered **(D)** individuals. **(E)** Clustering based on S1-IFN-γ-producing T cell average and neutralizing titer average 15 days after the second BNT162b2 dose, when the vaccine-elicited immune response peaked, in naïve individuals. Green arrows represent the time of SARS-CoV-2 infection. Black crosses represent SARS-CoV-2-infected subjects after mRNA vaccination. Horizontal bars and whiskers represent median values and interquartile ranges, respectively. The significance between groups was determined using Mann-Whitney or Kruskal-Wallis tests, ns: not statistically significant, **p<0.01, ***p<0.001, ****p<0.0001.

Five naïve individuals had a SARS-CoV-2 infection 3 to 6 months after completing the standard 2-dose vaccination and another 12 naïve subjects suffered a breakthrough infection approximately 1 month after the boost, coinciding with the Omicron pandemic wave (**Figure 2A**). All breakthrough infections in naïve individuals ranged from asymptomatic to mild COVID-19 (WHO ordinal scale of 0-2). In contrast, no breakthrough infection was recorded in the COVID-19 recovered individuals, neither after the second or the third dose (**Figure 2B**). As expected, none of the individuals who remained naïve developed M- or N-specific T cells during follow-up (**Figure 2C**), while the absence of breakthrough infections in recovered individuals could be confirmed by the lack of any boost in the specific T cell response against M or N proteins (**Figure 2D**). Of note, all SARS-CoV-2 recovered individuals maintained cellular response against M and/or N above the positivity threshold during follow-up (**Figure 2D**), which meant recovered subjects exhibited detectable virus-specific memory T cells up to 22 months after infection, coinciding with the post-boost sample. Recovered subjects were protected against breakthrough infection compared to naïve (0/17 versus 17/60, Fisher’s test, p=0.017). Demographic and immunological characteristics of subjects infected after vaccination are shown in **Table 1**. None of the analysed immune parameters were consistently low in infected compared to uninfected individuals. Interestingly, a cluster analysis in naïve individuals based on the mean peak cellular and neutralizing response after vaccination showed that subjects who developed both high T cell (>700 S1 IFN-γ sfu/10^6^ PBMCs) and neutralizing (>1/1206 IU/ml) response, were protected against breakthrough infection (**Figure 2E**). These analyses suggest that individuals with a lower-than-average cellular or neutralizing response may benefit from the administration of a third booster dose.

**Table 1:** Characteristics of SARS-CoV-2 infected individuals after mRNA vaccination.

Subject	Sex^a^	Age(years)	Time from last dose to infection	Peak T cell^b^ (sfu/10^6^PBMCs)	T cell prior to infection^c^(sfu/10^6^PBMCs)	Peak IgG^b^ (OD ratio)	IgG prior to infection^c^(OD ratio)	Peak Nabs^b^ (IU/ml)	Nabs prior to infection^c^ (IU/ml)
1	F	39	96 days	158	NA	10.1	NA	470	NA
2	F	25	154 days	1598	237	25.6	11.0	1543	338
3	M	22	163 days	1987	730	19.8	8.7	1111	297
4	F	51	170 days	577	60	9.4	6.2	612	109
5	F	41	178 days	308	280	12.6	9.7	1075	340
6	F	52	17 days	387	225	18.0	7.8	2580	244
7	F	33	18 days	663	88	28.2	8.2	2120	178
8	F	37	18 days	610	223	21.9	3.25	NA	NA
9	F	61	19 days	847	413	18.9	7.1	924	269
10	F	37	20 days	605	170	22.1	8.2	1111	222
11	M	30	21 days	119	108	27.9	10.3	2835	297
12	F	56	26 days	548	153	12.2	6.83	NA	NA
13	F	45	28 days	313	203	10.7	5.3	358	169
14	F	34	29 days	658	197	22.6	4.6	2611	178
15	M	44	31 days	915	297	23.9	8.9	1659	271
16	F	25	32 days	1672	1017	24.1	8.1	1622	362
17	F	43	34 days	213	43	20.8	8.4	1467	410
Mean Breakthrough Infection				716	277	19.3	7.7	1473	263
Mean Naïve During Follow-up				711	258	17.0	7.1	1206	206
Mean Naïve Cohort				700	256	18.0	7.0	1206	206

a F, Female; M, Male.

b Peak response was achieved 15 days after the second BNT162b2 dose.

c Subjects 1-5 were infected after the 2^nd^ dose, their timepoint prior to infection was 3 months.

Subjects 6-17 were infected after the 3^rd^ dose, their timepoint prior to infection was 6 months.

**Figure 3:**
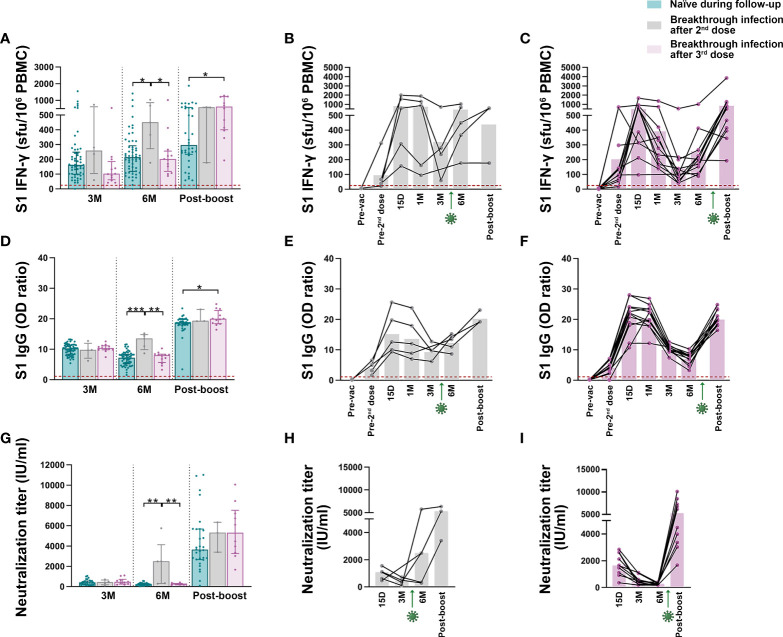
Effect of breakthrough infections on existing SARS-CoV-2 immunity. **(A)** Comparison of S1-IFN-γ-producing T cells among naïve individuals who remain naïve (in blue) and those with breakthrough infections after 2^nd^ (in grey) and 3^rd^ dose (in purple). Out of the 5 subjects who had SARS-CoV-2 infection after the 2^nd^ dose only 3 subjects received the third vaccine dose. **(B, C)** Longitudinal data on the dynamics of specific T cells in the 5 subjects infected by SARS-CoV-2 after the 2^nd^ dose **(B)** and in the 12 individuals infected after the 3^rd^ dose **(C)**. **(D)** Comparison of anti-S1IgG levels among SARS-CoV-2 naïve individuals and breakthrough infections after 2^nd^ and 3^rd^ dose. **(E, F)** Dynamics of antibody levels in subjects infected after the 2^nd^ **(E)** and the 3^rd^ dose **(F)**. **(G)** Comparison of neutralizing activity among SARS-CoV-2 naïve individuals and breakthrough infections after 2^nd^ and 3^rd^ dose. **(H, I)** Neutralizing activity in SARS-CoV-2 infected subjects after the 2^nd^ **(H)** and the 3^rd^ dose **(I)**. Green arrows represent the time of SARS-CoV-2 infection. Dashed lines represent the positivity cut-off. The significance between groups was determined using Mann Whitney test, *p<0.05, **p<0.01, ***p<0.001. (See **Figure 1** footnote for more detailed information).

Finally, regarding the effect of breakthrough infections on immunity, SARS-CoV-2 infections after the second or the third vaccine dose increased the S1-specific T cell response compared to individuals who remained naïve throughout the study (**Figures 3A–C**, **S5A–D**), and, as expected, also induced the development of IFN-γ T cells against SARS-CoV-2 M and N proteins (**Figures S5E, F**). Both total (**Figures 3D–F**) and neutralizing (**Figures 3G–I**) antibodies were also boosted after exposure to the virus.

## Discussion

There is currently the need to better understand the longevity of specific immune responses after SARS-CoV-2 infection and/or vaccination and to characterize the effect of booster doses on immunity in order to take informed decisions on the adequacy, and optimal interval, of successive vaccine doses. Our longitudinal analysis of cellular and humoral SARS-CoV-2-specific immunity elicited by mRNA vaccination showed the different dynamic and maintenance of virus-specific memory depending on whether or not a SARS-CoV-2 infection has been passed prior to vaccination. In addition, we describe in real-world conditions how a third mRNA vaccine dose not only boosted pre-existing SARS-CoV-2 humoral but also cellular immunity.

Similarly to previous reports we found robust antigen-specific cellular and humoral responses after 2-dose BNT162b2 vaccination schedule (5, 17, 39), which relates with a high effectiveness in preventing SARS-CoV-2 infection in the real world (1, 40, 41). However, a decrease in immunity was observed 1 month post-vaccination, and while T cells reached a plateau, antibodies continued to decline steadily. This waning immunity could cause, together with the emergence of viral variants, the observed decrease in vaccine effectiveness (13, 14, 41, 42). In our cohort there was a patient with a particularly poor cellular and humoral response to vaccination who was a 60-year-old man with a past history of alcohol abuse and currently in a good general condition under treatment with angiotensin-converting enzyme inhibitor, beta-blocker, disulfiram and gabapentin. This medical history could possibly explain his weak immune response; however, he remained naïve during follow-up.

So far, most studies have focused on the magnitude of the spike-specific antibody response or neutralizing titer following COVID-19 vaccination (4, 6, 8, 43–45). Our results showed that the number of S1-specific T cell clones remains stable from 3 to 6 months after the second BNT162b2 dose administration, confirming recent observations (7, 25). SARS-CoV-2 infection studies have demonstrated that T cell response is essential for viral clearance, may prevent infection without seroconversion, provides robust memory, and mediates recognition of viral variants (46). In our cohort, all individuals who had COVID-19 prior to vaccination maintained a cellular response against M and N above the positivity threshold 22 months post-symptom onset. This characterization of virus-specific T cell memory up to 22 months goes beyond previous studies that limited SARS-CoV-2-specific T cells determination up to 12 (47) and 15 months post-infection (48). In addition, to date, vaccine-elicited T cell responses remain capable of recognizing all known SARS-CoV-2 variants (25, 49). Altogether these data suggest that SARS-CoV-2-specific T cells could provide long-term protection against severe COVID-19 and death.

The strength and maintenance of IFN-γ-producing T cells and total and neutralizing antibodies from 1 to 6 months post-vaccination were significantly higher in SARS-CoV-2 recovered compared to naïve individuals. Recovered subjects present what is called “hybrid immunity”, which is developed by the combination of vaccination and natural infection, and results in more potent and long-lived immune response (50), partly due to a wider breadth for T cells and antibodies (51, 52). In fact, in our cohort none of the recovered subjects had a breakthrough infection during the 12-month follow up period. The protection shown by recovered subjects suggests that the design of vaccines that include antigens from viral proteins other than spike may complement and boost the immunity in already vaccinated population.

Administration of the third vaccine dose boosted humoral and cell-mediated immunity, which likely explains the findings that COVID-19 booster vaccination restores relative effectiveness to 90-95% against severe disease or death (27, 28, 53). The boost in T cells was more marked in recovered individuals, while the increase in humoral response, especially neutralizing antibodies, was remarkable in naïve subjects. The third dose produced a 20-fold increase in neutralization in naïve individuals and only then neutralizing antibodies titers were similar to those of recovered subjects. On the contrary, recovered individuals only had a modest antibody boost which suggests that pre-existing high levels of circulating antibodies may limit the effect of vaccine boosting (54). SARS-CoV-2 naïve individuals clearly benefited from the booster dose because it greatly enhanced their neutralizing capacity, while the benefit of booster vaccination in recovered individuals is questionable, as already indicated (55). These results may help to better identify target populations that could benefit most from booster doses and to design new fit vaccine strategies.

In our cohort, none of the analysed immune parameters, namely specific T cells, antibodies or neutralizing titers, associated individually with protection from breakthrough infection. It was the combination of a high cellular and neutralizing response after vaccination that protected against breakthrough infection in naïve individuals. Our previous work showed that the development of early and coordinated cellular and humoral responses upon SARS-CoV-2 infection led to a mild course of the disease (19). Patients who initially responded to infection by producing a large amount of antibodies tended to develop severe COVID-19. Similarly, the increased production of antibodies after vaccination observed in some naïve subjects did not associate with protection. These findings highlight the relevance of monitoring SARS-CoV-2-specific cellular immune responses, and not only antibody levels, as a correlate for protection after infection and/or vaccination.

A limitation in the search for protection immune correlates is the lack of standardized assays, especially for the measuring of specific cellular responses and neutralizing activity. Mangia *et al.* recently suggested that simultaneous neutralizing antibody titers <1/20, binding antibody levels <200 BAU/ml and IFN-γ <1,000 mIU/ml could identify subjects at risk of breakthrough infections (56). However, the definition of specific thresholds for risk or protection will not be useful until standardized assays to analyse specific T cell memory and neutralization are developed. In addition, the percentage of women is higher than that of men, which may be a limitation of the study. However, we found no association between sex and strength of the cellular or humoral immune response.

In summary, our work highlights that both specific T cells and neutralizing antibodies are important to prevent SARS-CoV-2 infection and to reduce severity in breakthrough infections. The third dose significantly increased cellular and humoral immunity against SARS-CoV-2, by enhancing neutralizing capacity especially in naïve individuals and specific T cells mainly in recovered subjects. In order to prevent future infections and severe cases of COVID-19, it would be advisable the administration of booster doses to all naïve individuals and the inclusion of viral antigens other than spike in new mRNA vaccine designs.

## Data availability statement

The raw data supporting the conclusions of this article will be made available by the authors, without undue reservation.

## Ethics statement

The Institutional Review Board approved the study (21/039 and 21/056). The patients/participants provided their written informed consent to participate in this study.

## Author contributions

PA-V, RL-G, RD, and EP-A conceived and designed the study. PA-V, MC-L, CG-C, MM-B, LS-P, and RL-G recruited participants and collected clinical data. PA-V, MC-L, AU-R, CG-C, MM-B, LS-P, JL, and NL performed the experimental work. PA-V, MC-L, AU-R, JL, and MF performed the statistical analysis. PA-V, RL-G, and EP-A wrote the manuscript. All authors contributed to interpretation of the data. All authors revised the manuscript and approved the final version before submission.

## Funding

This study was supported by the Instituto de Salud Carlos III, Spanish Ministry of Science and Innovation (COVID-19 research call COV20/00181) — co‐financed by the European Development Regional Fund “A way to achieve Europe”, Operative Program Intelligent Growth 2014-2020, and by Comunidad de Madrid (INMUNOVACTER REACT-UE) to EP-A. Instituto de Investigación Carlos III grant FIS PI2100989 and the European Commission Horizon Europe (project EPIC-CROWN-2 ref 101046084) to RD. RL-G holds a research contract “Rio Hortega” (CM19/00120) from the Instituto de Salud Carlos III, Spanish Ministry of Science and Innovation. MC-L holds a predoctoral fellowship (FPU19/06393) from the Spanish Ministry of Science and Innovation.

## Acknowledgments

We would like to thank all participants, nurses and medical colleagues who contributed to the study.

## Conflict of interest

The authors declare that the research was conducted in the absence of any commercial or financial relationships that could be construed as a potential conflict of interest.

## Publisher’s note

All claims expressed in this article are solely those of the authors and do not necessarily represent those of their affiliated organizations, or those of the publisher, the editors and the reviewers. Any product that may be evaluated in this article, or claim that may be made by its manufacturer, is not guaranteed or endorsed by the publisher.

## Abbreviations

COVID-19, Coronavirus disease 2019; EMPS, Epitope Mapping Peptide Set; IFN-γ Interferon-γ; IL-2, Interleukin-2; IU/ml, International Units per ml; IQR, Interquartile range; M, Membrane; NT50, Neutralizing titer 50; N, Nucleocapsid; NA, Not assessed; PBMCs, Peripheral blood mononuclear cells; RSV, Relative spot volume; SARS-CoV-2, Severe acute respiratory syndrome coronavirus 2; Sfu, Spot forming units.
