# Supplementary material for: Cellular and humoral immune responses and breakthrough infections after three SARS-CoV-2 mRNA vaccine doses
Source: Front Immunol. 2022 Aug 17;13:981350. doi: 10.3389/fimmu.2022.981350 (PMC9428395; doi:10.3389/fimmu.2022.981350)
Supplement: Supplementary file 1 [file DataSheet_1.docx]

**SUPPLEMENTARY FIGURES**


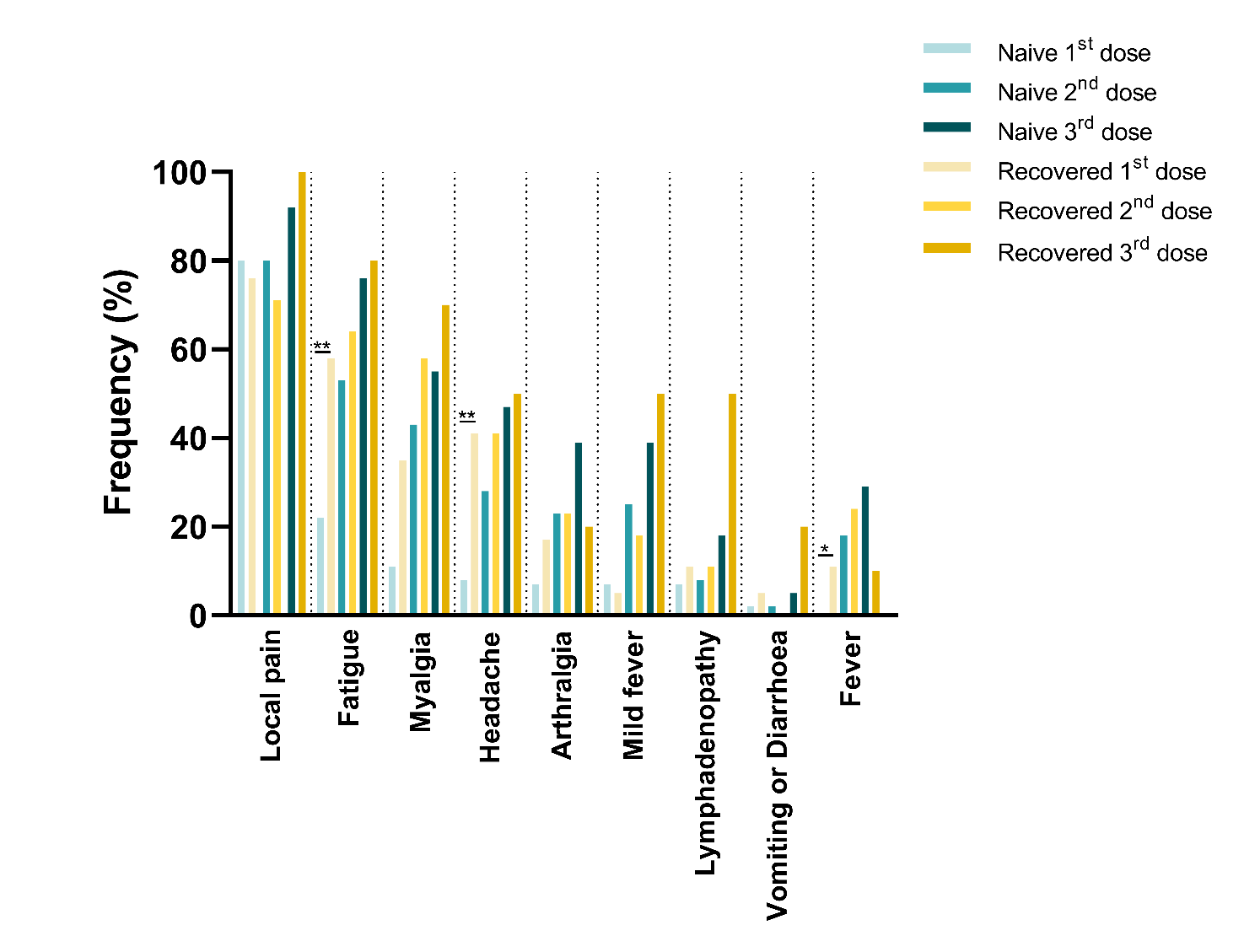


**Figure S1. Frequency of side effects after the three vaccine doses according to prior SARS-CoV-2 infection status.** The most common side effect after vaccination was local pain followed by fatigue and myalgia. Frequency of side effects increased with repeated exposure to SARS-CoV-2, including the initial exposure to the virus in recovered subjects. The significance between groups was determined using Mann Whitney test, *p<0.05, **p<0.01, ***p<0.001, ****p<0.0001. (See Fig. 1 footnote for more detailed information).


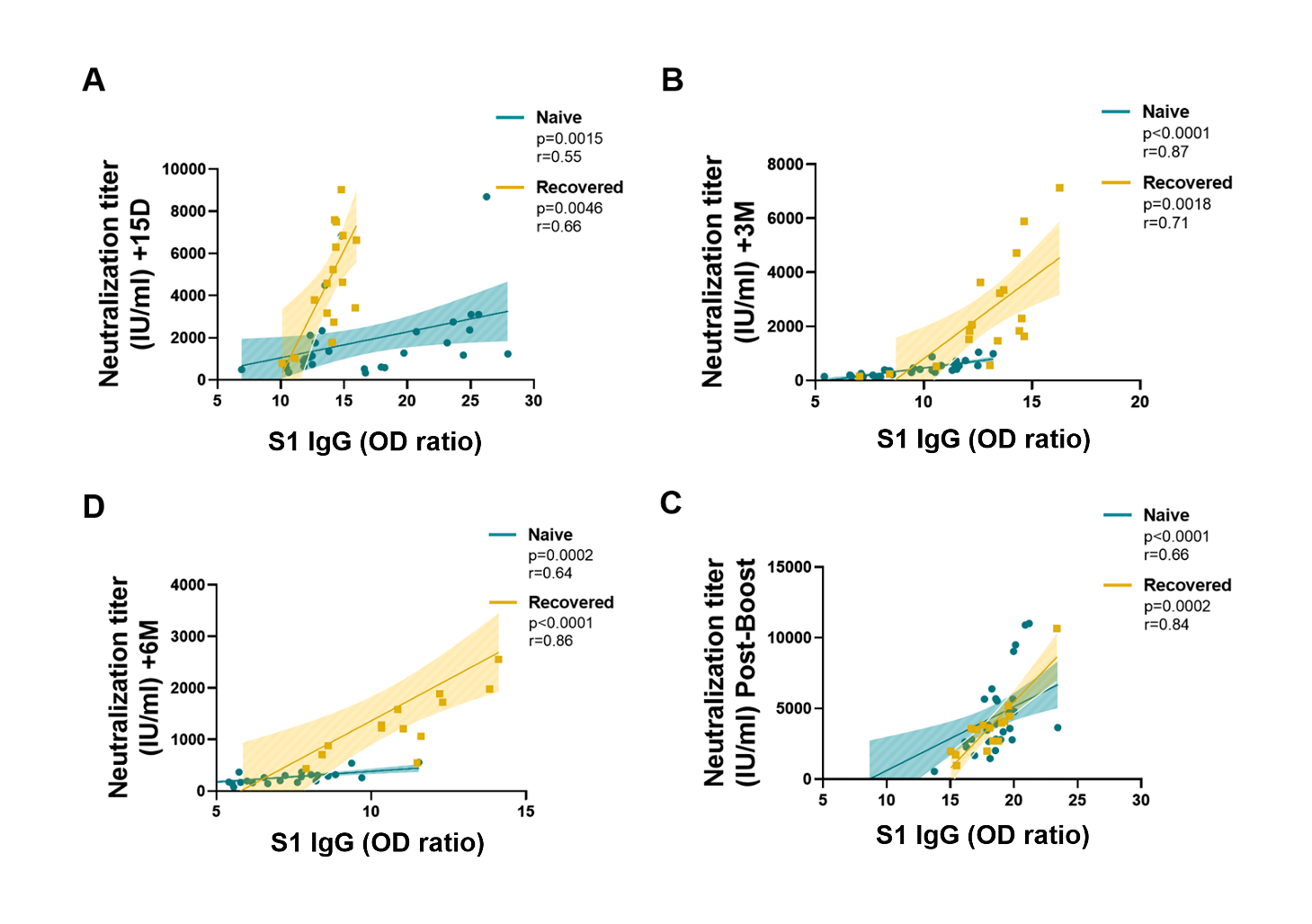


**Figure S2.** **Positive correlation between anti-S1 IgG and neutralization in SARS-CoV-2 naïve and recovered individuals.** Correlation between IgG and neutralization titers (IU/ml) measured 15 days (**A**), 3 months (**B**) and 6 months (**C**) after the administration of the first BNT162b2 dose and one month after the third mRNA-1273 boost dose (**D**). Linear regressions were performed using Spearman’s rank test.


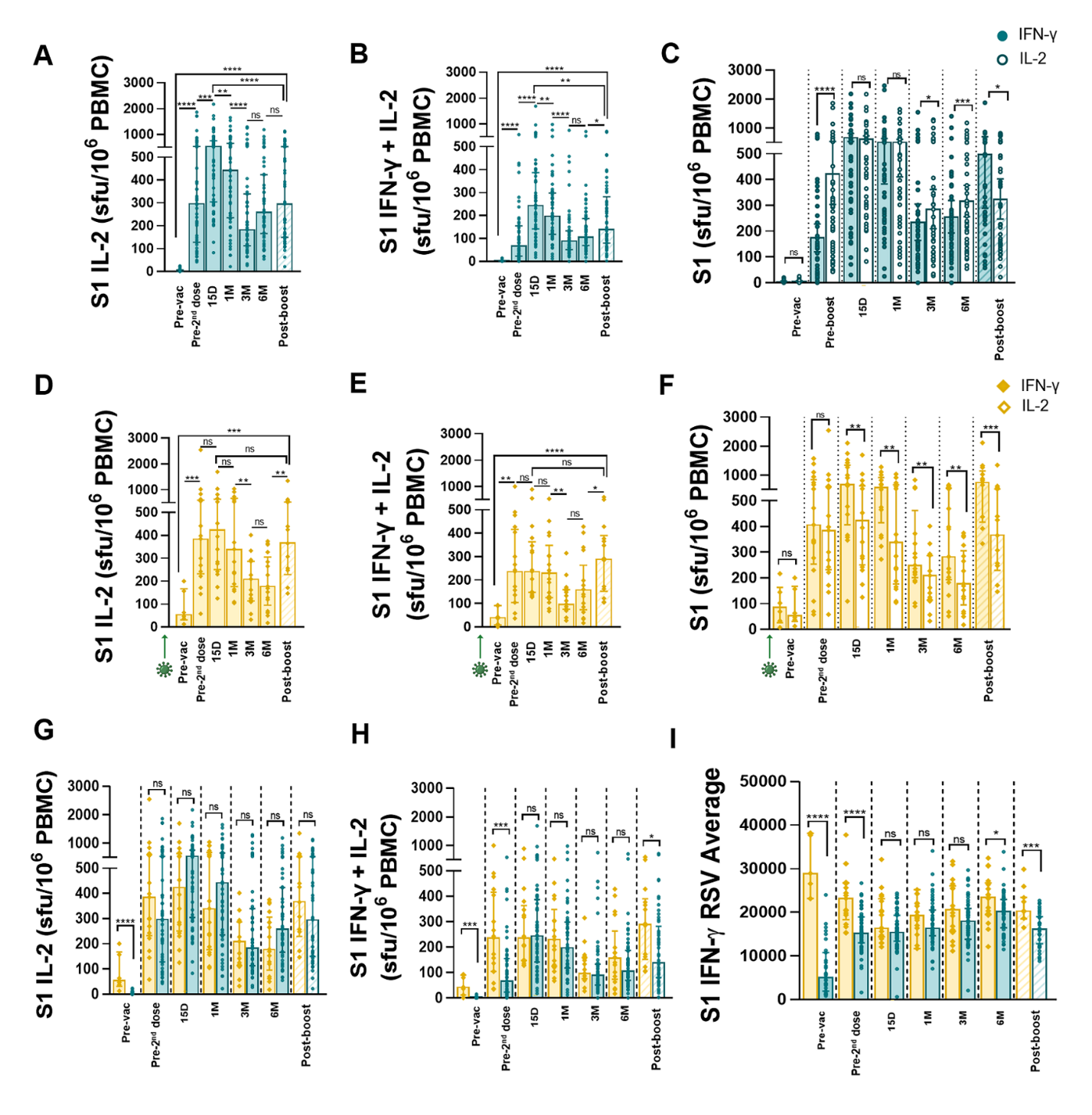


**Figure S3. Dynamics of SARS-CoV-2-specific IFN-γ and IL-2 cellular response after mRNA vaccination.** **A-B**) IL-2 (A) and bifunctional IFN-γ+IL-2 (B) T cell responses against S1-SARS-CoV-2 protein according to the sample collection in SARS-CoV-2 naïve individuals. **C**) Comparison of S1-IL-2-producing and S1-IFN-γ-producing specific T cells in naïve individuals. **D-E)** IL-2 (D) and bifunctional IFN-γ+IL-2 (E) T cell responses against S1-SARS-CoV-2 protein in SARS-CoV-2 recovered individuals. **F**) Comparison of S1-IL-2-producing and S1-IFN-γ-producing specific T cells in recovered individuals. **G-H)** Comparison of IL-2 (G) and bifunctional IFN-γ+IL-2 (H) S1-specific T cell responses between SARS-CoV-2 naïve and recovered individuals. **I)** Comparison of IFN-γ RSV after mRNA vaccination between SARS-CoV-2-naïve and -recovered individuals. Green arrows represent the time of SARS-CoV-2 infection. Horizontal bars and whiskers represent median values and interquartile ranges, respectively. The significance between groups was determined using Mann Whitney, Wilcoxon signed rank or Kruskal-Wallis tests, *p<0.05, **p<0.01, ***p<0.001, ****p<0.0001.


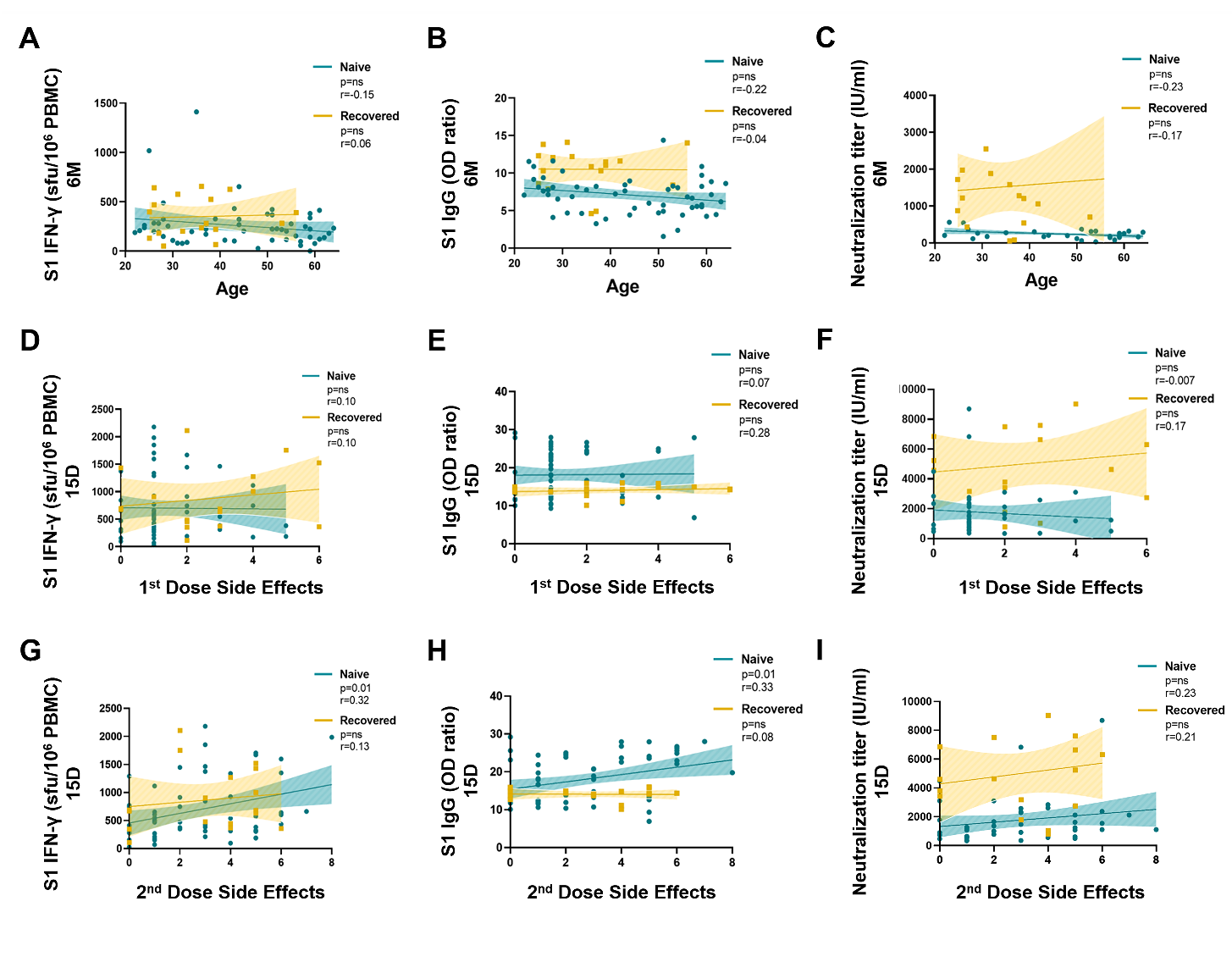


**Figure S4. Maintenance of SARS-CoV-2-specific adaptive immune response and correlations between the number of side effects suffered after mRNA vaccination and the magnitude of cellular and humoral responses in naïve and recovered COVID-19 individuals. A-C)** Correlation between age and S1 IFN-γ-producing T cells (A), anti-S1 IgG (B) and neutralizing antibodies (C) six-months after the second BNT162b2 dose administration. **D-F)** Correlation between the number of side effects reported after the first BNT162b2 dose administration and the S1-IFN-γ-producing T cells (D), anti-S1 IgG (E) and neutralizing antibodies (F) at peak levels. **G-I)** Correlation between the number of side effects after the second BNT162b2 dose administration and the S1-IFN-γ-producing T cells (G), anti-S1 IgG (H) and neutralizing antibodies at peak levels. Linear regressions were performed using Spearman’s rank test.


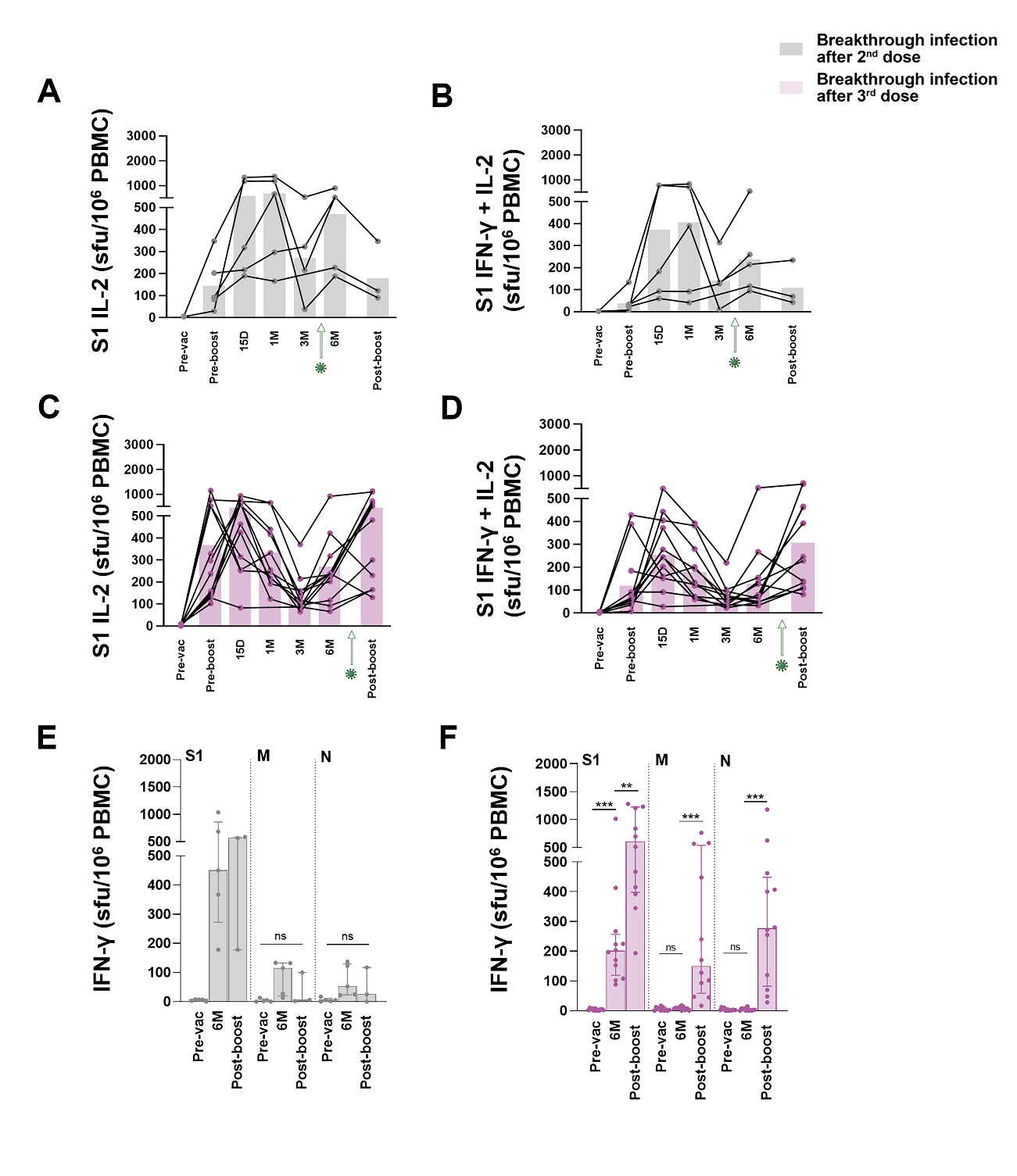


**Figure S5. Dynamics of SARS-CoV-2-specific cellular response after mRNA vaccination and breakthrough infections.** IL-2 and bifunctional IFN-γ+IL-2 T cell responses in individuals infected by SARS-CoV-2 after the second (**A-B**) or third (**C-D**) vaccine dose. SARS-CoV-2-specific IFN-γ-producing T cell responses reactive to the S1, M and N proteins in individuals infected by SARS-CoV-2 after the second (**E**) or third (**F**) vaccine dose. Horizontal bars and whiskers represent median values and interquartile ranges, respectively. The significance between groups was determined using Mann Whitney or Kruskal-Wallis tests, *p<0.05, **p<0.01, ***p<0.001, ****p<0.0001.
